# The Surface Polarized Graphene Oxide Quantum Dot Films for Flexible Nanogenerators

**DOI:** 10.1038/srep32943

**Published:** 2016-09-06

**Authors:** Liangbin Liu, Yafei Cheng, Lili Zhu, Shuit-Tong Lee, Fan Liao, Mingwang Shao

**Affiliations:** 1 Institute of Functional Nano and Soft Materials (FUNSOM), Jiangsu Key Laboratory for Carbon-based Functional Materials and Devices & Collaborative Innovation Center of Suzhou Nano Science and Technology, Soochow University, Suzhou, Jiangsu 215123, P. R. China

## Abstract

Abundant disorderly-distributed surface functional groups, such as hydroxyl, carboxyl, ether and amino groups, endow an isolated graphene oxide quantum dot (GOQD) the polar property due to the symmetry breaking, although the aggregated counterparts present no polarization owing to the random orientation. Here, flexible polarized films were fabricated using aggregated GOQDs with the assistance of external electric fields and their polarization was confirmed with the electrostatic force microscopy and polarization-electric field hysteresis loop. Such polarized GOQD films may induce charges under externally applied deformation. Here, we fabricated nanogenerators based on the films, which gave out an average current value of 0.12 *μ*A and an average voltage value of 12 V under a mechanical force of 60 N. This work has proposed a convenient electric-field-assisted method to give the nanomaterials new functions, which can be generalized to other materials and found applications in various fields.

In 2006, a ZnO-nanowire based nanogenerator was first proposed by Wang *et al*.^1^. After that, numerous nanogenerators are developing rapidly and vigorously due to their easy-fabricating, environmental friendly and practicability. Different active materials used in these generators, such as BaTiO_3_^2^, polyvinylidene fluoride^3^, silicon nanowires^4^,^5^, and graphene oxide^6^. The power generation mechanisms include piezoelectricity^1^,^5^,^7^,^8^, triboelectricity^9^,^10^,^11^,^12^,^13^,^14^,^15^,^16^, electret^4^,^17^, pyroelectricity^18^,^19^, and electrostatics^20^. Among the various mechanisms, the piezoelectric one occupied a special position because a small force may generate a huge potential difference^21^.

Graphene shows no piezoelectricity for its centrosymmetry, although graphene and its ramifications have attracted tremendous interest, which are treated as the most appropriate material for the future electronic applications^22^,^23^,^24^,^25^,^26^. Normally, graphene oxide quantum dots (GOQD) own plenty of polar functional groups, such as hydroxyl, carboxyl, ether and amino groups, disorderly distributed on the edge of the basal plane. Yet, aggregated GOQDs show no apparent polarizability due to their disorder orientation.

In this work, we are surprised to find that the aggregated GOQD film showed a remnant polarization in the polarization-electric field hysteresis loop. Therefore, an external electric field was introduced to orientate GOQD films, making the functional groups orientated along the direction of the electric field. The polarized GOQD film possessed plenty of surface charges, as confirmed by the electrostatic force microscopy (EFM). The GOQD film with surface charges can be employed as an active material in the fabrication of the nanogenerators on a flexible polyethylene terephthalate (PET) substrate, which may convert the mechanic energy into electricity.

## Results

**Figure 1:**
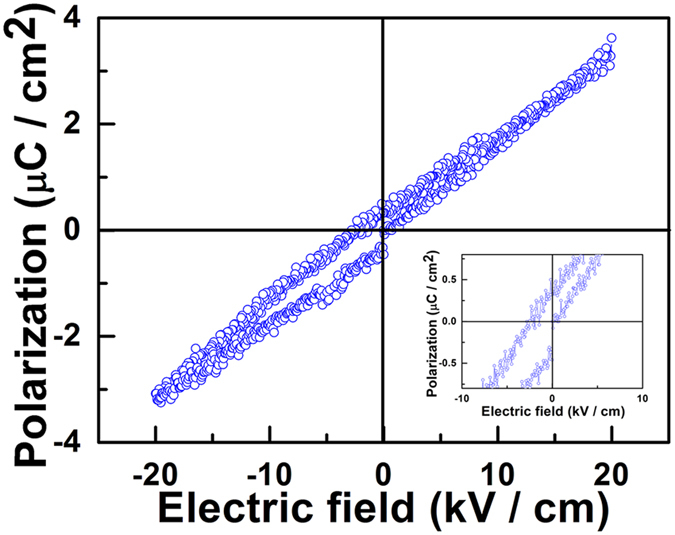
The polarization-electric field hysteresis loop of the GOQD film, the inset is the enlargement of the central area from −10 to 10 kV/cm.

The adopted GOQDs here were prepared by a hydrothermal method. These QDs, with the average diameter of 2.3 nm (Figures S1 and S2, Supplementary Information), were fabricated into GOQD films, which was examined by the polarization-electric field hysteresis loop (Fig. 1), showing a remnant polarization of 0.4 *μ*C/cm^2^.

Considering the polarizability of the un-polarized GOQD film, a polarized GOQD film could be fulfilled by an external electric field of 250 V/cm during the drying process. The texture structure of the polarized and un-polarized GOQD films fabricated on the ITO substrate was investigated with XRD patterns. As shown in Figure S3 (Supplementary Information), the un-polarized GOQD film only shows a weak (001) peak at 11.5°, while the polarized GOQD film has not only a strong (001) peak, but also a clear (002) peak at 23.4° (marked with black diamonds). These peaks were attributed to the regular packing of GOQD films. And the appearance of (002) peak reveals that the polarized GOQD film has a better orientation than the un-polarized one. The other diffraction peaks (marked with black dots) may be attributed to the ITO substrate (JCPDS 89-4596).

**Figure 2:**
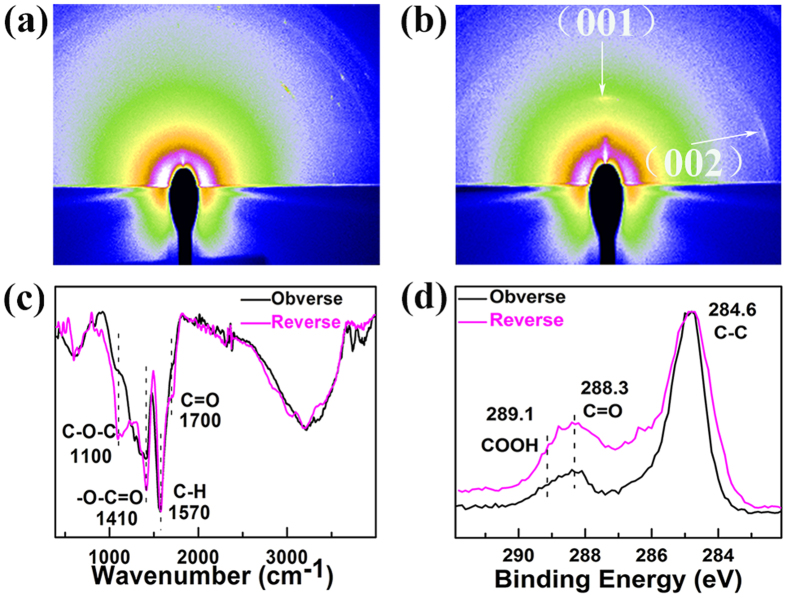
The synchrotron radiation based grazing incidence two-dimensional X-ray diffraction patterns of (**a**) un-polarized GOQD film, and (**b**) polarized GOQD one. (**c**) FTIR and (**d**) high-resolution XPS C1s core spectra of the obverse and reverse sides of the polarized GOQD film.

In order to further investigate the texture structure, the synchrotron radiation based grazing incidence two-dimensional X-ray diffraction patterns of the un-polarized and polarized GOQD films were collected. The pattern of the un-polarized GOQD film (Fig. 2a) shows no obvious diffraction rings, while clear (001) and (002) special diffraction rings of layered structure exist in the pattern of the polarized GOQD film (Fig. 2b), which has indicated better orientation of the polarized GOQD film than the un-polarized one.

The polarization was considered to be closely related to the functional groups of the GOQDs. Therefore, a detailed FTIR characterization (Fig. 2c) about the functional groups of two sides of the GOQD film was conducted (the reverse side is defined as the side connected to the substrate). The intense vibrational bands at 1570 cm^−1^ may be attributed to the stretching vibrations of C-H bond. Peaks at 1700, 1410, and 1100 cm^−1^ were assigned to the C = O, -O-C = O, and -O-C-O vibrations, respectively^27^. These nucleophilic functional groups are enriched on the reverse side under the electric field, so the signals are much stronger in the reverse side than the obverse one. Judged from the FTIR, the polarization direction of the film is from the obverse side to the reverse one.

The XPS provides another strong technique to analyze the surface functional groups of the obverse and reverse sides of the polarized GOQD film. The C1s core spectra of both sides of the polarized GOQD film are exhibited in Fig. 2d, which can be classified into three peaks matching to C-C (284.6 eV), C = O (288.3 eV), and COOH (289.1 eV), respectively. With normalization of the C-C peak at the binding energy of 284.6 eV, both peaks of C = O and COOH show higher intensities in the reverse side of the polarized GOQD film than those in the obverse side, which further testifies that the nucleophilic polarity functional groups are accumulated in the reverse side after the polarization in the electric field.

**Figure 3:**
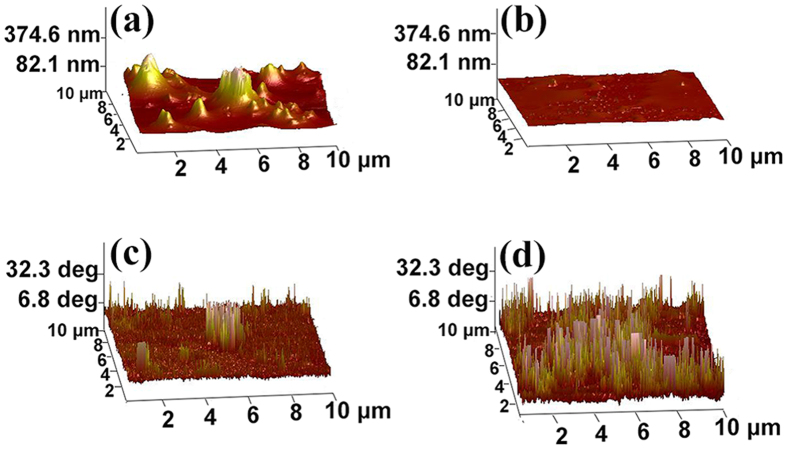
AFM profiles: surface height of (**a**) the un-polarized GOQD film and (**b**) polarized GOQD one; EFM images: surface phase image of (**c**) the un-polarized GOQD film and (**d**) polarized GOQD one.

The information of the morphology and polarization of two kinds of films were characterized with AFM and EFM (Fig. 3). Figure 3a,b explicitly show the morphology difference of these two GOQD films on a 10 × 10 *μ*m^2^ scanning area. The surface of the un-polarized GOQD film is rough with the surface roughness of 68.1 nm, while that of the polarized one is only 7.9 nm. The smooth surface of the polarized GOQD film may be associated with the external electric field: there are numerous gas bubbles in the film during the drying process; with the help of the electric field, the gas bubbles were deformed, then escaped from the GOQD dispersion, resulting in a smooth surface. When no electric field was exerted on the GOQD film, the bubbles were still in a spherical shape and difficult to release from the dispersion, leaving a rough surface of the film.

The corresponding surface charge density has been observed from the phase patterns in Fig. 3c,d. The polarized GOQD film shows obvious response comparing with the un-polarized one in the EFM images. The average phases of the un-polarized and polarized film are 5.4 and 28.9 degree, respectively. The distinction of the phase values of the GOQD films manifests that the applied external electric field successfully alters the distribution of the surface charge. Furthermore, the phase pattern of the polarized GOQD film is shown a positive response as a whole, which testifies that the polarization direction is perpendicular to the substrate from top to bottom.

**Figure 4:**
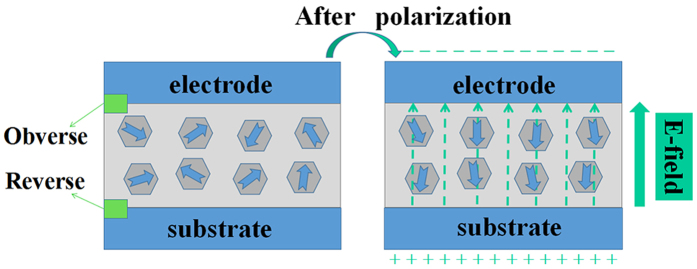
The schematic diagram about the polarization process with the electric field assistance: (**a**) random arrangement of the functional groups, and (**b**) ordered arrangement of the functional groups under the electric field.

Combining with the information from EFM, FTIR and XPS, the polarization of the electric-field-treated GOQD film is schematically shown in Fig. 4. The dipole moments in the GOQDs (marked with arrows) are randomly distributed before applying an electric field. In the electric-field-assisted drying process, the nucleophilic functional groups were attracted to the substrate; at the same time, the dipole moments were aligned to point to the substrate, finally leading to a polarized to GOQD film. The surface charges may keep for a long time due to the long-lasting dielectric relaxation phenomenon, allowing the polarized film to be applied in the generators.

**Figure 5:**
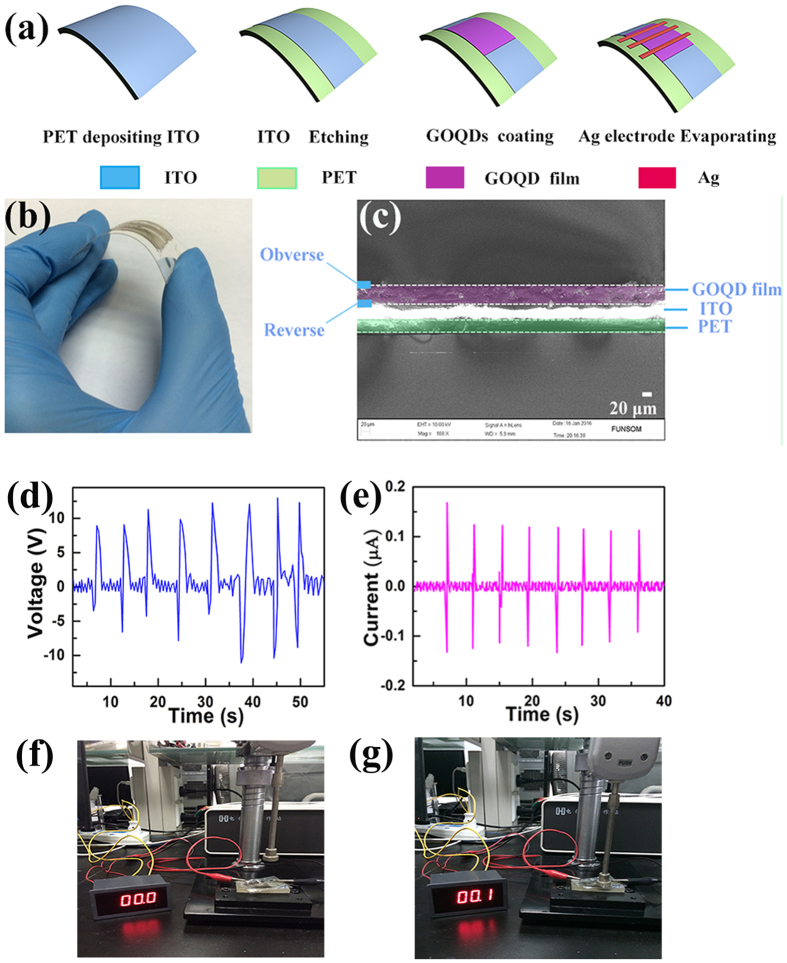
(**a**) The fabrication process and device structure of the flexible GOQD film nanogenerator; (**b**) the photograph of the flexible nanogenerator; (**c**) SEM image illustrating the cross-section view of the nanogenerator; (**d**) The output voltage value of a polarized GOQD nanogenerator; (**e**) The output current value of a polarized GOQD nanogenerator; and a microammeter showing the induced current value with (**f**) no force, and (**g**) an external force of 60 N.

The fabrication process of the GOQD nanogenerator was exhibited in Fig. 5a. The ITO deposited flexible PET substrate was selected as an electrode. Then, the GOQD dispersion was transferred onto the center of the ITO. Meanwhile, an electric field of 250 V/cm was applied during the drying process in the air. After the film was formed, Ag electrodes were evaporated on the top of the film with a mask to fabricate GOQD nanogenerators with a flat-panel structure.

An optical photograph of the GOQD nanogenerator (Fig. 5b) reveals that the device is flexible and robust. It may be bent with various directions and can bear 2000 N twisting force without being damaged. The SEM image illustrates the cross-section view of the nanogenerator in Fig. 5c. Three layers can be clearly observed as they are piled up neatly.

The working mechanism of the polarized GOQD film-based nanogenerator (Figure S4, Supplementary Information) is as follows: When a force was applied on the nanogenerator, a potential between the silver and ITO electrodes is produced as for the induced charges in the GOQD film, and the external free charges will move to the electrodes to equilibrate the induced potential, expressed as the detection of the current signals (Figure S4b, Supplementary Information). When the force on the nanogenerator is released, the potential between the two electrodes disappears. The external charges on the two electrodes move back, resulting in the current signals in an opposite direction, as shown in Figure S4c (Supplementary Information).

The output voltage of the polarized GOQD nanogenerator was shown in Fig. 5d with an average value of 12 V. Figure 5e is the output current value of a polarized GOQD nanogenerator under an external force of 60 N, with the average current being 0.12 *μ*A. Another nanogenerator was fabricated using the un-polarized GOQD film as active materials for comparison, no obvious output current (Figure S5, Supplementary Information) being measured when a force of 60 N is applied. The different values of currents in Fig. 5e and Figure S5 (Supplementary Information) indicated that the electric-field-induced polarization has positive effects on the output currents of the nanogenerators.

A microammeter was also applied to demonstrate the induced current as shown in Fig. 5f,g: The display screen just shows 0 *μ*A without the exerted force; and an obvious current value of 0.1 *μ*A is shown when a force of 60 N is acted with a pressure gauge.

In order to investigate its potential in practical applications, the GOQD nanogenerators were employed to excite a light emitting diode (LED), as shown in the video in the Supplementary Information. It is clearly observed that the LED was lighted when a force was applied on the device. Figure S6 (Supplementary Information) displayed the schematic circuit diagram for the lighting LED system.

## Discussion

The value of the remnant polarization (0.4 *μ*C/cm^2^) in Fig. 1 is a strong evidence for the polarizability of GOQD film, which indicates that GOQD films can be polarized using the electric-field-assisted method.

XRD patterns, AFM and EFM images indicate the differences between the polarized and un-polarized GOQD films. Polarized GOQD films show better orientation (Fig. 2a,b and S3, Supplementary Information), smoother surface (Fig. 3a,b) and larger surface charge density (Fig. 3c,d). The surface roughness of polarized GOQD films is less than one-eighth of that of the un-polarized ones; and the average phase of the former is 5 times larger than that of the latter. FTIR (Fig. 2c) and XPS (Fig. 2d) spectra indicate the differences between the number of functional groups on both sides of the polarized GOQD films, with the nucleophilic functional groups enriched on the reverse side under the electric field, showing the polarization direction of the film from the obverse side to the reverse one (Fig. 4).

The nanogenerators may output an average voltage value of 12 V (Fig. 5d) and current value of 0.12 *μ*A (Fig. 5e) under a mechanical force of 60 N, which was large enough to drive a LED light, as shown in the video in the Supplementary Information.

In order to clarify the polarization of the polarized GOQD nanogenerator, the switching polarity test was performed with changing the electrodes of connecting to the nanogenerator. The inverse current signals shown in Figure S7 (Supplementary Information) verified that the charges were induced by the polarized GOQD nanogenerator.

The electric-field-assisted method offers a new route to fabricate polarized films. We hope that the performed work on polarizability of GOQDs and the corresponding flexible nanogenerator with an easy fabrication way will be served as an exemplar for other nanomaterials.

## Methods

### Synthesis and Characterization

#### Materials

PET coated with a layer of indium tin oxide (ITO) was purchased from the Aldrich Company. Pyrene was purchased from J&K Chemical Science and Technology Ltd. All the materials were used without further purification.

#### The synthesis of GOQDs

First 1,3,6-trinitropyrene was obtained by filtering the nitration production of pyrene under refluxing and stirring in HNO_3_ at 80 °C for 12 h. Then 0.15 g 1,3,6-trinitropyrene was ultrasonic dispersed in 0.2 M NaOH solution (35 mL) for 2 h to form a suspension. The suspension was transferred into a 50 mL Teflon-lined autoclave and heated at 180 °C for 10 h. After cooling to room temperature, a 0.22 *μ*m microporous membrane was utilized to filter the insoluble impurities. Finally, a dialysis bag was employed to dialyze the filtrate to obtain the GOQDs.

#### Fabrication of the polarized and un-polarized GOQD film

GOQD dispersion (2 mL, 1 mg/mL) was added on the center area (2 cm × 1 cm) of ITO or Si substrates with size of 3 cm × 1 cm. An external electric field of 250 V/cm was applied employing ITO or Si substrates as the positive electrode when the GOQD film was drying in the air. The same process was performed in the fabrication of the un-polarized GOQD film, except that no external electric field was applied when the film was drying in the air.

#### Fabrication of the GOQD film nanogenerators

A flexible PET substrate with a layer of ITO was cut in the size of 3 cm × 4 cm and treated by UV-Ozone radiation for 15 min. Then 2 mL 1 mg/mL GOQD dispersion was added dropwise on the ITO layer to form a film with size of 2 cm × 1 cm. The dispersion was dried in the air when an electric field of 250 V/cm was applied employing ITO coated PET as the positive electrode. After the film was formed, Ag electrodes with thickness of 60 nm were evaporated on the top of the film by utilizing a shadow mask to fabricate GOQD nanogenerators by an electron beam evaporator (PVD75).

#### Pressure operation experiment

In the output current and voltage test, a pressure gauge (HANDPI NK-100) equipped with a slider (diameter: 1.5 cm) was applied to export the mechanical force of 60 N with a frequency of 0.2 Hz. A microammeter was also applied to demonstrate the output current with the same pressure gauge as shown in Fig. 5f,g.

#### Characterization

The morphology of the section view was investigated on a Zeiss Supra 55 field scanning electron microscopy (SEM). The output voltage and current of the nanogenerators were measured with a Keithley 4200-SCS system of semiconductor characterization. Fourier transform infrared (FTIR) measurements were performed using a Bruker FTIR spectrometer equipped with ATR accessory. The X-ray photoelectron spectroscopy (XPS) was conducted with a KRATOS AXIS Ultra DLD spectrometer. Two-dimensional X-ray diffraction pattern was carried out on the BL14B1 beamline in the Shanghai Synchrotron Radiation Facility (SSRF). The ferroelectric tester (Radiant 609B-3, USA) was performed to measure the polarization-electric field hysteresis loop of the un-polarized GOQD films at room temperature. The atomic force microscopy (AFM, Bruker Dimension Icon) and electrostatic force microscopy (EFM, Bruker Dimension Icon) were applied to characterize the surface topographic height and surface phase pattern of the GOQD film, respectively. The X-ray diffraction (XRD, Philips X’pert PRO MPD diffractometer) with Cu Kα radiation (λ = 0.15406 nm) was performed to characterize the texture structure of the films. The transmission electron microscopy (TEM) images were recorded using a FEI Tecnai F20 transmission electron microscope with accelerating voltage of 200 kV.

## Additional Information

**How to cite this article**: Liu, L. *et al*. The Surface Polarized Graphene Oxide Quantum Dot Films for Flexible Nanogenerators. *Sci. Rep.* **6**, 32943; doi: 10.1038/srep32943 (2016).

## Supplementary Material

Supplementary Information

Supplementary Movie S1
