# Supplementary material for: The Surface Polarized Graphene Oxide Quantum Dot Films for Flexible Nanogenerators
Source: Sci Rep. 2016 Sep 6;6:32943. doi: 10.1038/srep32943 (PMC5011759; doi:10.1038/srep32943)
Supplement: Supplementary Information [file srep32943-s1.pdf]

Supporting Information

The Surface Polarized Graphene Oxide Quantum Dot Films for Flexible Nanogenerators

Liangbin Liu, Yafei Cheng, Lili Zhu, Shuit-Tong Lee*, Fan Liao, Mingwang Shao*

Institute of Functional Nano and Soft Materials (FUNSOM), Jiangsu Key Laboratory for Carbon-based Functional Materials and Devices& Collaborative Innovation Center of Suzhou Nano Science and Technology, Soochow University, Suzhou, Jiangsu 215123, P. R. China

1. TEM images of GOQDs

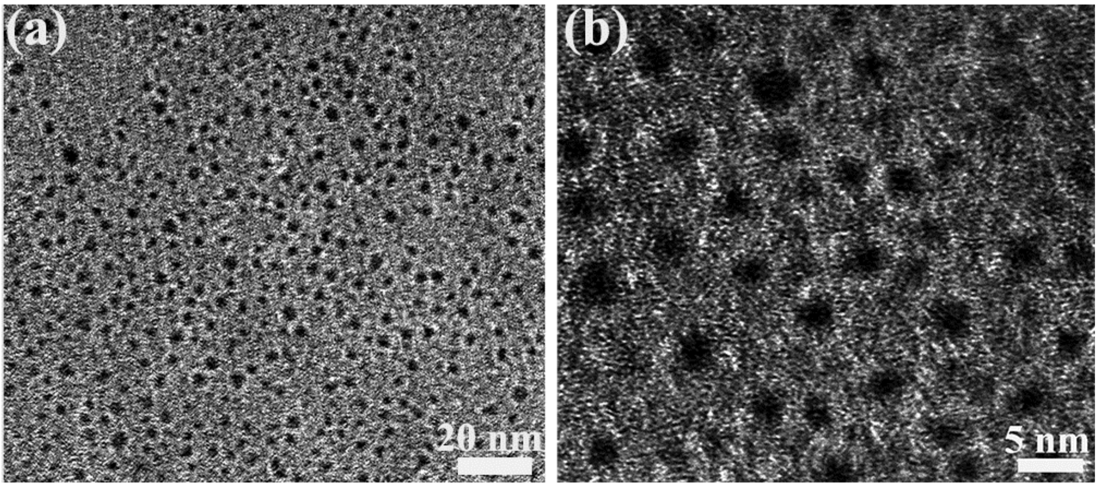

Figure S1. TEM images of the GOQDs in (a) small and (b) large magnification.

2. The size distribution histogram of GOQDs

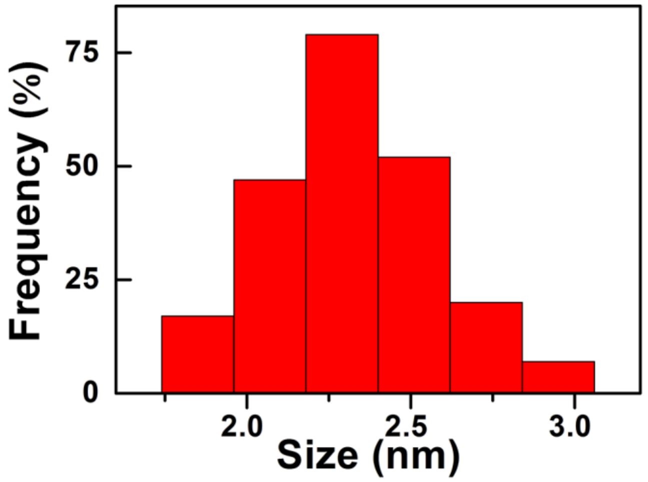

Figure S2. The size distribution histogram of GOQD from TEM images based on 220 dots, showing the average diameter of 2.3 nm.

3. XRD patterns of the polarized and un-polarized GOQD films

The (001) and (002) diffraction peaks (Curve a, marked with black diamonds) are attributed to the layered structure of the polarized GOQD film and only the (001) one can be observed for the un-polarized GOQD film. Other peaks marked with black dots may be indexed as the ITO substrate (JCPDS 89-4596).

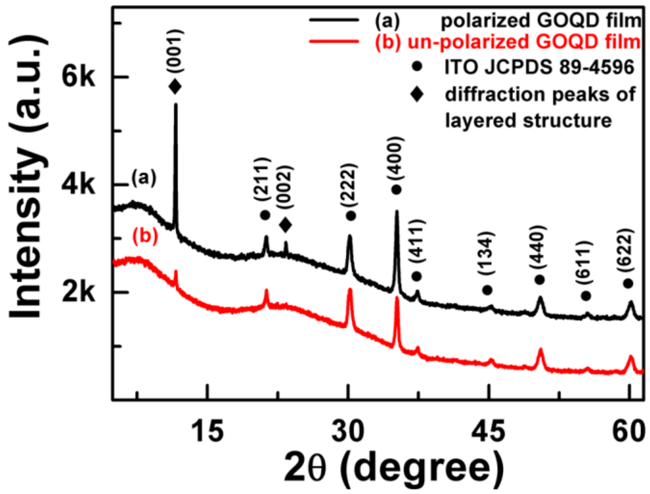

Figure S3. XRD patterns of (a) the polarized and (b) un-polarized GOQD film depositing on the ITO substrate.

4. Working mechanism of the polarized GOQD film-based nanogenerator

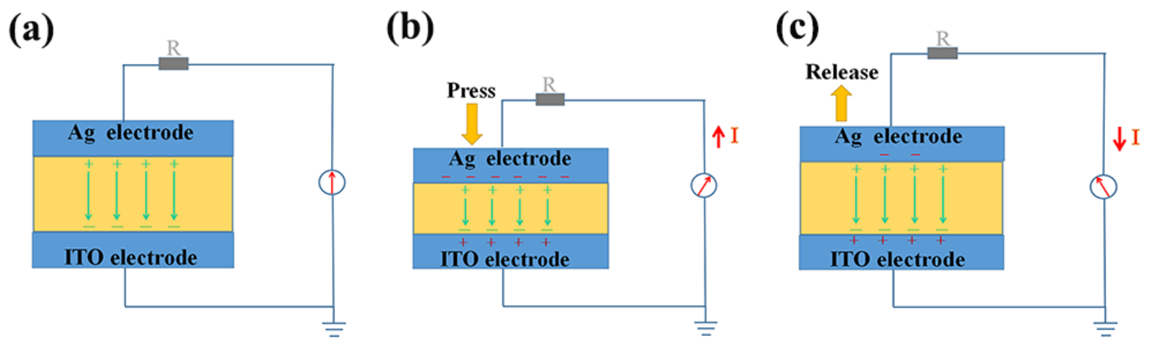

Figure S4. The diagram of the equivalent circuit of the polarized GOQD film based nanogenerator.

5. Output current of an un-polarized GOQD film based nanognerator

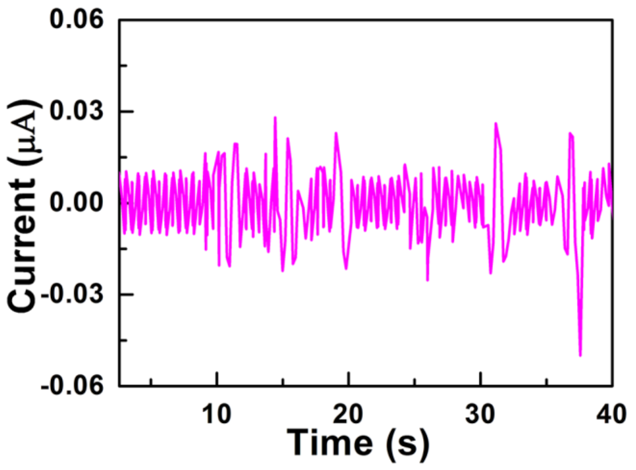

Figure S5 The output current value of an un-polarized GOQD film based nanognerator.

6. Schematic circuit diagram of the lighting LED system

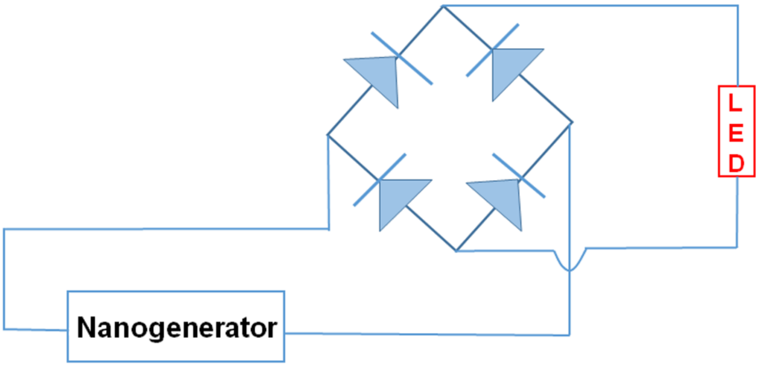

Figure S6. Schematic circuit diagram of the lighting LED system.

7. Switching polarity test of a polarized GOQD nanogenerator

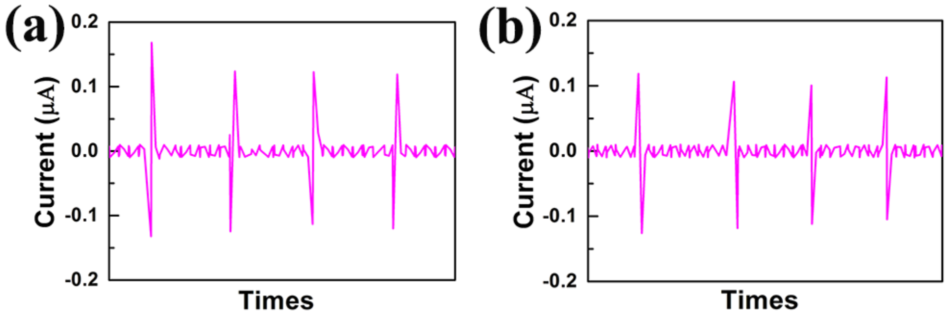

Figure S7. (a) and (b) Switching polarity test of a polarized GOQD nanogenerator.

8. Video Legends

The supplementary video illustrates a red flash LED in response to the output electricity converted from external mechanical energy with a nanogenerator.
